# Molecular and biophysical mechanisms behind the enhancement of lung surfactant function during controlled therapeutic hypothermia

**DOI:** 10.1038/s41598-020-79025-3

**Published:** 2021-01-12

**Authors:** C. Autilio, M. Echaide, A. Cruz, C. García-Mouton, A. Hidalgo, E. Da Silva, D. De Luca, Jorid B. Sørli, J. Pérez-Gil

**Affiliations:** 1 Department of Biochemistry and Molecular Biology and Research Institute “Hospital 12 de Octubre (imas12), Faculty of Biology, Complutense University, Jose Antonio Novais 12, Madrid, Spain; 2 National Research Centre for the Working Environment, Copenhagen, Denmark; 3 Department of Environmental Engineering, Technical University of Denmark, Kgs. Lyngby, Denmark; 4 Division of Pediatrics and Neonatal Critical Care, “A.Béclère” Medical Center, Paris Saclay University Hospitals, APHP, Paris, France; 5 Physiopathology and Therapeutic Innovation Unit-INSERM U999, South Paris-Saclay University, Paris, France

**Keywords:** Biochemistry, Biophysics, Physiology, Medical research, Molecular medicine

## Abstract

Therapeutic hypothermia (TH) enhances pulmonary surfactant performance in vivo by molecular mechanisms still unknown. Here, the interfacial structure and the composition of lung surfactant films have been analysed in vitro under TH as well as the molecular basis of its improved performance both under physiological and inhibitory conditions. The biophysical activity of a purified porcine surfactant was tested under slow and breathing-like dynamics by constrained drop surfactometry (CDS) and in the captive bubble surfactometer (CBS) at both 33 and 37 °C. Additionally, the temperature-dependent surfactant activity was also analysed upon inhibition by plasma and subsequent restoration by further surfactant supplementation. Interfacial performance was correlated with lateral structure and lipid composition of films made of native surfactant. Lipid/protein mixtures designed as models to mimic different surfactant contexts were also studied. The capability of surfactant to drastically reduce surface tension was enhanced at 33 °C. Larger DPPC-enriched domains and lower percentages of less active lipids were detected in surfactant films exposed to TH-like conditions. Surfactant resistance to plasma inhibition was boosted and restoration therapies were more effective at 33 °C. This may explain the improved respiratory outcomes observed in cooled patients with acute respiratory distress syndrome and opens new opportunities in the treatment of acute lung injury.

## Introduction

Therapeutic hypothermia (TH) is used as an effective method to limit brain injury after certain types of cardiac arrest and perinatal asphyxia, by reducing patient body temperature from 37 to 33.5 °C^1,2^. Early studies also suggest some possible benefits in cooled patients suffering of acute respiratory distress syndrome (ARDS)^3–6^. ARDS accounts for 10% of all cases in intensive care units worldwide. Mortality remains at 30–40% and no effective treatments are available to date^7^. Primary ARDS occurs after a direct insult to the lung, whereas secondary ARDS is triggered by an extra-pulmonary pro-inflammatory event^7–9^. Theoretically, TH may be useful to treat primary but might be harmful in secondary ARDS^10,11^. Conversely, during primary ARDS the injury is at first confined to the lung, resulting in lower compliance and more severe surfactant dysfunction than in the secondary form of the syndrome^7–9^.

During ARDS, surfactant function is impaired in several ways^12,13^. Protein-rich oedema fluid, high levels of cholesterol and secretory phospholipase A2 (sPLA_2_) can all inhibit surfactant activity directly and enhance the production of reactive oxygen species (ROS). Lung surfactant is dramatically needed to reduce surface tension under the expiration-mediated shrinking of the alveolar air–liquid interface. The precise proportions of dipalmitoylphosphatidylcholine (DPPC), unsaturated phospholipids and hydrophobic surfactant proteins B (SP-B) and C (SP-C) are essential for its function in a healthy lung^12^. However, oedema proteins, such as albumin, increase in ARDS patients^14^ and may compete with surfactant membranes for the air–liquid interface. The resulting interfacial steric barrier affects the in vitro surfactant adsorption and the reduction in surface tension under compression^15^. Beyond the physiological range, the drastic rise in cholesterol compared to the saturated lipid amount leads to surfactant membranes fluidification^12^. This impairs the ability of clinical and native surfactants to decrease surface tension upon breathing-like cycles^16,17^. Besides, methyl-β-cyclodextrin can ex vivo overcome this cholesterol-mediated inhibition in a mice model of ARDS^18,19^. PLA_2_ targets and hydrolyses the condensed domains of phosphatidylcholine monolayers with a crumbling effect at the air–liquid interface^20^. Consistently, high levels of the secretory form of the enzyme was detected in ARDS patients along with changes in surfactant phospholipid composition and surface tension properties^21^. As a result, surfactant amount and performance are reduced and the work of breathing increases upon ARDS^13^.

Interestingly, cooled animals with different types of lung failure showed better respiratory mechanics and reduced lung tissue inflammation^22–24^. Moreover, reports on cooled asphyxiated neonates with and without neonatal ARDS due to meconium aspiration describe an improvement in surfactant function^25–27^, pulmonary inflammation^27^, oxygenation and some clinical outcomes such as reduction of respiratory support and hospital stay^25,28^. The biological mechanisms underlying this benefit are not totally clear. DPPC turnover did not change in cooled neonates^29^, but a reduction in sPLA_2_ activity has been reported^30^.

However, several biophysical mechanisms may also contribute to surfactant performance under cooling. Drastic changes in body temperature leads to adaptive variations in both surfactant structure and composition to maintain proper interfacial properties in hibernating animals^31,32^. Besides, the surface activity of rabbit lung extracts tends to improve decreasing temperature from 40 to 15 °C^33^.

It is well known that the organization and physical properties of lipid membranes, like those in lung surfactant, can be influenced by shifting temperatures towards their melting points^34^. Cooling to values below 30 °C affects lung surfactant performance in the presence of plasma compounds^35^. However, the reduction from 37 to 33 °C may have a different effect, as it is still above surfactant melting temperature (≈ 32 °C)^34^.

To the best of our knowledge, no detailed in vitro studies have investigated surfactant biophysical properties under TH. Here, we analyse for the first time both the interfacial structure and the composition of lung surfactant films subjected to compression at 33 °C compared with 37 °C. We also describe enhanced surfactant function and resistance to inhibition by plasma under cooling, as a well as a more efficient restoration of function as a consequence of further surfactant supplementation. Finally, we investigate the molecular mechanisms governing this enhanced performance and propose a feasible biophysical model.

## Results

### Moderate hypothermia enhances surfactant activity in a concentration-dependent manner

**Figure 1:**
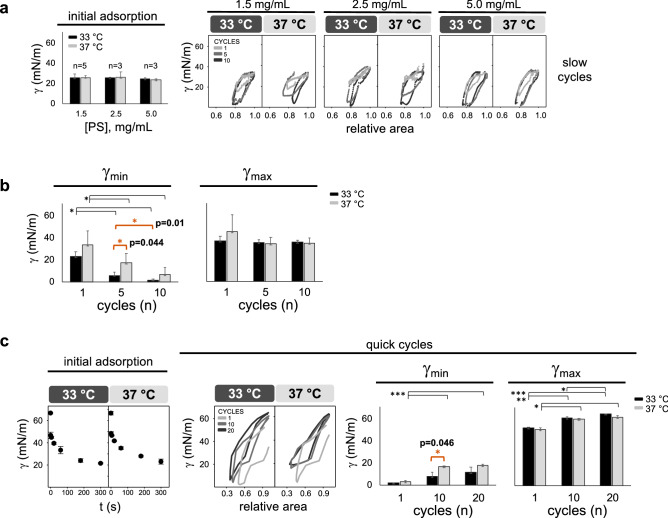
Changes in PS activity depending on temperature and concentration under slow and quick compression-expansion cycling. (**a**) CDS experiments under increasing concentration of PS (from 1.5 to 5 mg/mL) at 33 °C and 37 °C. On the left, surface tension values (γ, mN/m) reached by PS 10 s after the buffer deposition on top of the pedestal (initial adsorption). On the right, one typical replicate of γ-∆area isotherms representative of the ten slow compression-expansion cycles (2 cycles/min, shrinking the interface area by 20%, 0.2 cm^2^/min). (**b**) Minimum and maximum surface tensions reached during cycles 1, 5 and 10, testing surfactant at limiting concentration (1.5 mg/mL, n = 5). (**c**) CBS experiments, using a PS concentration of 1.5 mg/mL at 33 °C and 37 °C. On the left, γ values reached during 5 min (300 s) of PS adsorption after injecting material onto the bubble (initial adsorption). In the middle, one representative replicate (n = 3) of γ-∆area isotherms under 10 quick compression-expansion cycles (20 cycles/min). On the right, minimum and maximum surface tensions reached during cycles 1, 5 and 20. Black and light-grey bars represent experiments performed at 33 °C and 37 °C, respectively. Means and standard deviation of at least three replicates are shown. Compression-expansion cycles are depicted in grey scale. Horizontal lines represent statistical comparisons. Two-way ANOVA test followed by post-hoc test = (a) initial adsorption: n. s.; (b) γ_min_: temperature (p = 0.002, D.F. = 1, F = 11.6) and cycles (p < 0.001, D.F. = 2, F = 30.2), γ_max_: n. s.; (c) initial adsorption: n. s.; γ_min_: temperature (p = 0.001, D.F. = 1, F = 20.2) and cycles (p < 0.001, D.F. = 2, F = 40.2), γ_max_: temperature (p = 0.003, D.F. = 1, F = 13.4) and cycles (p < 0.001, D.F. = 2, F = 180.1). *p < 0.05 and > 0.01, **p ≤ 0.01 and > 0.005, ***p ≤ 0.005. The exact p values of the most relevant *post-hoc* tests are indicated. The corresponding comparison bars and symbols are highlighted in orange color. *PS* purified porcine surfactant, *γ* surface tension, *min* minimum, *max* maximum, *n. s.* not significant, *D.F.* degrees of freedom.

The activity of purified porcine surfactant (PS) was tested at different concentrations (1.5, 2.5 and 5 mg/mL) under slow (2 cycles/min) and quick (20 cycles/min) dynamic cycles by Constrained Drop Surfactometer (CDS) and Captive Bubble Surfactometer (CBS), respectively (Fig. 1). PS initial adsorption did not change among conditions (Fig. 1a), whereas dynamic activity significantly depended on sample concentration.

At limiting concentration (1.5 mg/mL), PS significantly reduced surface tension after 5 slow cycles at 33 °C, whereas it needed 10 cycles at 37 °C (Fig. 1a,b). This trend was also evident at a PS concentration of 2.5 mg/mL. Using the latter condition, there was a significant difference in γ_min_ comparing cycle 5 and 10 for material tested at 37 °C, but it was not the case for PS assayed at 33 °C (Supplementary Fig. S1a). However, this trend was not present at higher concentration (5 mg/mL) (Supplementary Fig. S1b).

Similar results were obtained in CBS experiments. At limiting concentration, γ_min_ is significantly lower after 10 quick cycles at 33 °C compared with 37 °C. Consistently, the minimum surface tension was significantly higher along cycles at 37 °C. At the same time, the maximum surface tension significantly rose upon the interfacial dynamics irrespective of the experimental temperature (Fig. 1c). This is probably related to the highly diluted surfactant used for the experiments and the resulting easy loss of material from the air–liquid interface under compressions.

### Moderate hypothermia increases the size of condensed domains in surfactant films, promoting the exclusion of unsaturated phospholipids upon breathing

**Figure 2:**
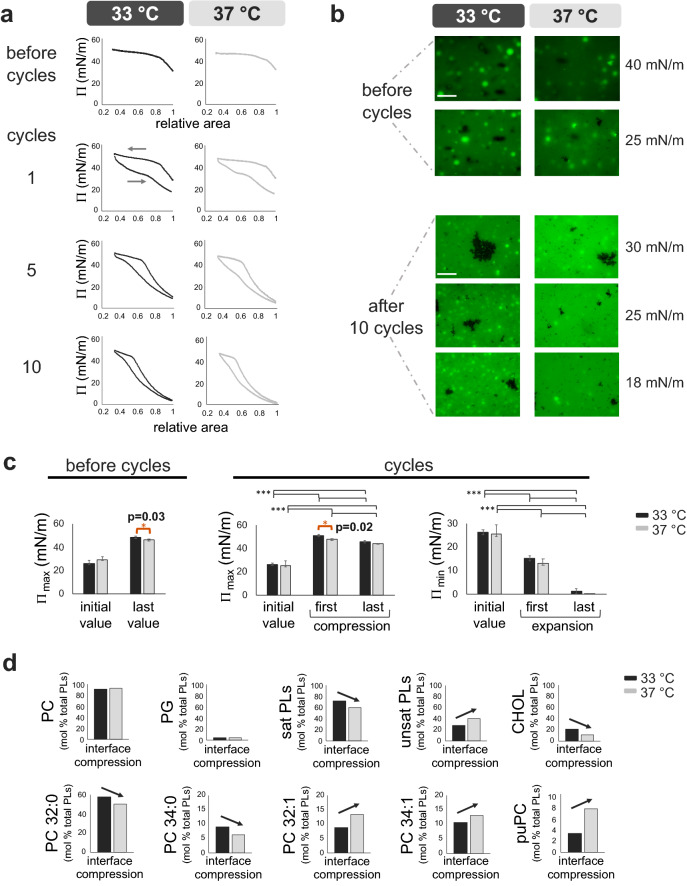
Temperature-dependent changes in PS activity, structure and composition in a Langmuir–Blodgett trough. (**a**) One representative replicate (n = 5) of Π-Δarea isotherms before and during 10 slow compression-expansion cycles of the interface (0.3 cycles/min, ≈65 cm^2^/min, reducing the interface area by ≈65%) at 33 °C and 37 °C. Around 50 μg (at 5 mg/mL) of PS was added at the interface before starting the experiment. Cycles 1, 5 and 10 are shown. (**b**) Epifluorescence analysis (n = 2) of PS lateral structure upon slow interface compressions (25 cm^2^/min) before and after 10 quicker compression-expansion cycles (65 cm^2^/min). PS was previously doped with the fluorescent probe BODIPY-PC (1% mol/mol). Upon compression, clusters of condensed lipids (enriched in saturated PL species) excluding the bulky fluorescent probe are visible as black domains against a green background (which represents the mixture of more expanded and disordered lipids and probe). Brilliant green spots, probably representing tridimensionally excluded structures, are also visible. The lateral structure of PS is shown before reaching the equilibrium plateau at several surface pressures. White scale bars, 100 μm. For each image, histogram stretching has been performed to enhance contrast without deleting pixel data. (**c**) On the left, the initial and the maximum surface pressures obtained before and after the first compression (25 cm^2^/min). On the right, the initial surface pressure obtained before starting the experiment together with the maximum and minimum surface pressures during the 1st and 10th compression-expansion cycles (65 cm^2^/min). Mean and SD of 5 replicates are shown. (**d**) Lipidomic analysis of PS proximally associated to the surface during compression of the interface (25 cm^2^/min) at 33 °C or 37 °C. The interface recollected from 5 replicates were pooled per condition and analysed by lipidomic analysis. All lipid species were detectable. Each lipid species was normalized with respect of the total amount of PLs and reported as percentage. Black and light-grey colors represent experiments performed at 33 °C and 37 °C, respectively. Black horizontal lines represent statistical comparisons. Black arrows highlight the variation in PS lipid species at 33 °C compared with 37 °C. Paired t test = Π_max_ before cycles: p = 0.029, t = 3.3, D.F. = 4. Two-way ANOVA test followed by post-hoc test = Π_max_ during cycles: temperature (p = 0.025, D.F. = 1, F = 5.7) and cycles (p < 0.001, D.F. = 1, F = 593); Π_min_ during cycles: temperature (p < 0.001, D.F. = 1, F = 375.5) and cycles (p < 0.001, D.F. = 2, F = 83.6). *p < 0.05 and > 0.01, ***p ≤ 0.005. The exact p values of the most relevant *post-hoc* tests are indicated. The corresponding comparison bars and symbols are highlighted in orange color. *Π* surface pressure, *max* maximum, *min* minimum, *PC* phosphatidylcholine, *PLs* phospholipids, *PG* phosphatidylglycerol, *sat* saturated, *unsat* unsaturated, *CHOL* cholesterol, *puPC* polyunsaturated phosphatidylcholine, *D.F.* degrees of freedom.

The activity and structure of PS were tested in a Langmuir–Blodgett trough (Fig. 2) before and after 10 compression-expansion cycles (0.3 cycles/min). PS activity was slightly better at 33 °C compared with 37 °C. Maximum surface pressures were significantly higher (minimum surface tensions significantly lower) at 33 °C compared with 37 °C. This occurred both before and in the first cycle of the experiments (Fig. 2a,c).

Upon cycling, the plateau during expansions was visible at higher surface pressures at 33 °C than 37 °C (Fig. 2a). This suggested that the compressed phase was more stable at 33 °C while the material tended to relax quicker at physiological temperature. Moreover, under compression of the air–liquid interface, a higher amount of surfactant seems to be excluded at 37 °C than at 33 °C and not replaced during the subsequent expansion. This results in a tendency to increase minimum surface pressures at 33 °C taking the last cycles into account (p = 0.09) (Fig. 2a,c).

Upon compression, and before further cycling, the condensed black domains at the surface films were rounder and less polymorphic than after compression-expansion cycling, as observed under epifluorescence microscopy (Fig. 2b). This result was detected regardless of the temperature, suggesting an intrinsically fluid character of the ordered phase before cycling, with a reduced contact perimeter with the coexisting surrounding disordered phases. On the contrary, a de-mixing of lipids, together with larger gel condensed phases, were observed after cycling, especially at 33 °C. These phases apparently contained smaller domains that were organized in clusters. Moreover, brilliant fluorescent spots were present at both temperatures and remained visible after cycles, especially at 33 °C. These probe-rich spots are likely areas of three-dimensional exclusion that appear bright due to the light scattering. Their apparent size was reduced after cycling associated with a lighter green liquid-expanded phase. Interestingly, due to the lower lipid miscibility at 33 °C than 37 °C, phase-coexistence was more evident under hypothermia temperature. This leads to larger green spots, presumably excluded from the interface, along with larger and darker condensed domains at 33 °C than 37 °C (Fig. 2b, Supplementary Fig. S2).

The composition of PS lipids that were proximally associated to the surface under compression was analysed by LC-HRMS (Supplementary Table S1). The percentage of saturated lipids (especially DPPC) and cholesterol increased at 33 °C compared with 37 °C, whereas the amount of unsaturated lipids, especially those with more than 2 double bonds (polyunsaturated phatidylcholine (puPC)), decreased (Fig. 2d).

### DPPC proportion, hydrophobic surfactant proteins and steric hindrance of excluded phospholipids contribute to improve surfactant activity at 33 °C

Several surfactant lipid mixtures (DPPC, palmitoyloleoylphosphatidylglicerol (POPG), palmitoyloleoylphosphatidylcholine (POPC) and dioleoylphosphatidylcholine (DOPC) at different proportions) with decreasing capability to pack under compression were combined with SP-B alone (1% total protein-to-lipid by weight) or with both SP-B and SP-C (2% total protein-to-lipid by weight). The combination of DPPC, POPC and POPG roughly simulates the composition of a typical lung surfactant in terms of saturated/unsaturated and zwitterionic/anionic phospholipid species. The presence of DOPC brings the contribution of phospholipids (PLs) with a large area per molecule, such as it is the case with polyunsaturated species.

**Figure 3:**
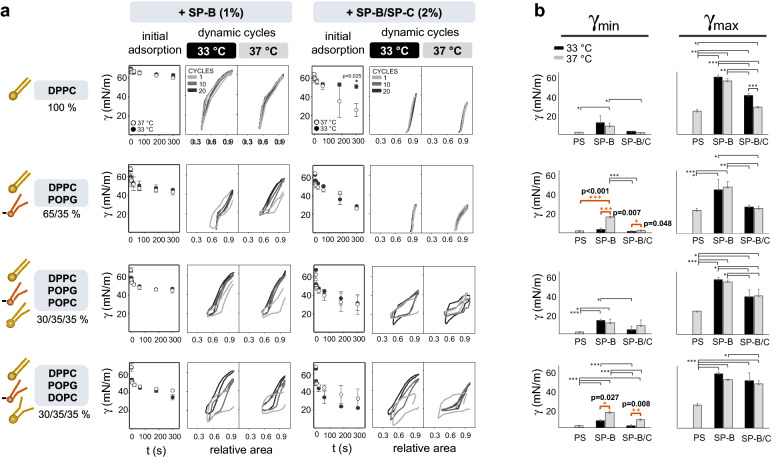
Contribution of lipid species and hydrophobic surfactant proteins in the temperature-mediated improvement of surfactant activity. Several surfactant lipid mixtures (DPPC, POPG, POPC and DOPC at different proportions) with decreasing capability to pack under compression were combined with purified porcine surfactant protein SP-B alone (1% w/w) or both SP-B and SP-C (2% total protein-to-lipid by weight). CBS experiments, using a concentration of 8 mg/mL of each lipid-protein mixture, were performed at 33 °C or 37 °C. (**a**) On the left, the mass percent of the lipids used in each experiment is shown. Drawings of the lipids are also shown to illustrate the different space occupied at the interface according to their unsaturation grade. Graphs of initial adsorption show means and SD of three replicates per condition. One representative replicate (n = 3) of γ-∆area isotherms under 10 quick compression-expansion cycles (20 cycles/min, 1.37 atm/s) is also shown. (**b**) Minimum and maximum surface tensions reached upon 20 dynamic cycles (n = 3), comparing each lipid–protein mixture to purified PS (8 mg/mL). Compression-expansion cycles are depicted in grey scale. Means and SD of three replicates are shown. Horizontal lines represent statistical comparisons. Paired t test = (a) γ(300 s) during initial adsorption: p = 0.025, D.F. = 2, t =  − 6.2. Two-way ANOVA test followed by post-hoc test = (b) first line, γ_min_: temperature (n. s.) and composition (p = 0.007, D.F. = 2, F = 8.4), γ_max_: temperature (p < 0.001, D.F. = 1, F = 26.4) and composition (p < 0.001, D.F. = 2, F = 150.6); second line, γ_min_: temperature (p < 0.001, D.F. = 1, F = 258.9) and composition (p < 0.001, D.F. = 2, F = 270.3), γ_max_: temperature (n. s.) and composition (p < 0.001, D.F. = 2, F = 22.4); third line, γ_min_: temperature (n. s.) and composition (p = 0.005, D.F. = 2, F = 9.4), γ_max_: temperature (n. s.) and composition (p < 0.001, D.F. = 2, F = 41.1); fourth line, γ_min_: temperature (p < 0.001, D.F. = 2, F = 168.9) and composition (p < 0.001, D.F. = 2, F = 160.9), γ_max_: temperature (n. s.) and composition (p < 0.001, D.F. = 2, F = 31.5). *p < 0.05 and > 0.01, **p ≤ 0.01 and > 0.005, ***p ≤ 0.005. The exact p values of the most relevant *post-hoc* tests are indicated. The corresponding comparison bars and symbols are highlighted in orange color. *γ* surface tension, *DPPC* dipalmitoylphosphatidylcholine, *POPG* palmitoyloleoylphosphatidylglicerol, *POPC* palmitoyloleoylphosphatidylcholine, *DOPC* dioleoylphosphatidylcholine, *PS* purified porcine surfactant, *SP-B* surfactant protein B, *SP-C* surfactant protein C, *min* minimum, *max* maximum, *n. s.* not significant, *D.F.* degrees of freedom.

The surface properties of each mixture were assayed under quick cycles (20 cycles/min) by CBS (Fig. 3a,b). No differences among 33 and 37 °C were noticed for PS at 8 mg/mL (data not shown). Thus, only results at physiological temperature (37 °C) for PS are shown and considered as control for optimal biophysical activity (γ_min_ < 2mN/m, γ_max_≈ 30 mN/m).

The presence of both SP-B and SP-C increased the adsorption and dynamic activity of all tested materials regardless of the temperature. Upon dynamic cycling, there was a significant reduction in minimal surface tensions at 33 °C compared with 37 °C when a high percentage of DPPC (65%) with respect to POPG (35%) was present (Fig. 3a, second line). This difference was more evident in the absence of SP-C, suggesting the importance of both hydrophobic proteins to exclude POPG from the interface under compression. Moreover, although both SP-B and SP-C were present (Fig. 3a, second line on the left), a trend to significance was observed at 33 °C for the reduction in area that is required to reach minimal surface tension: [13.6 (2.3)% at 33 °C vs 23.8 (2.2)% at 37 °C, p = 0.05].

Together with the DPPC content, the steric hindrance of excluded PLs under compression also enhanced surfactant activity at 33 °C. The difference among temperatures was significant in the presence of DOPC (Fig. 3a,b, fourth line). Conversely, the lipid-protein mixture containing POPC did not show any temperature-mediated changes in surface-active properties. PLs of this mixture probably did not mix well, leading to a marked variability among replicates in CBS and the co-existence of 2 peaks in Differential Scanning Calorimetry (DSC) (Supplementary Fig. S3a).

Finally, the dynamic properties of each mixture and PS were also compared (Fig. 3b). Regarding minimum surface tensions, PS activity was comparable to the activity of the mixture containing DPPC/POPG (65/35%, w/w) with SP-B alone or with SP-C. This result was more evident for material tested at 33 °C. Interestingly, the melting temperatures of these 2 mixtures (Supplementary Fig. S3a,b) were very close to TH: 32.4 (0.12) and 33.9 (0.06) °C.

### Moderate hypothermia decreases surfactant inhibition by plasma and enhances the restoration activity of therapeutic surfactants

**Figure 4:**
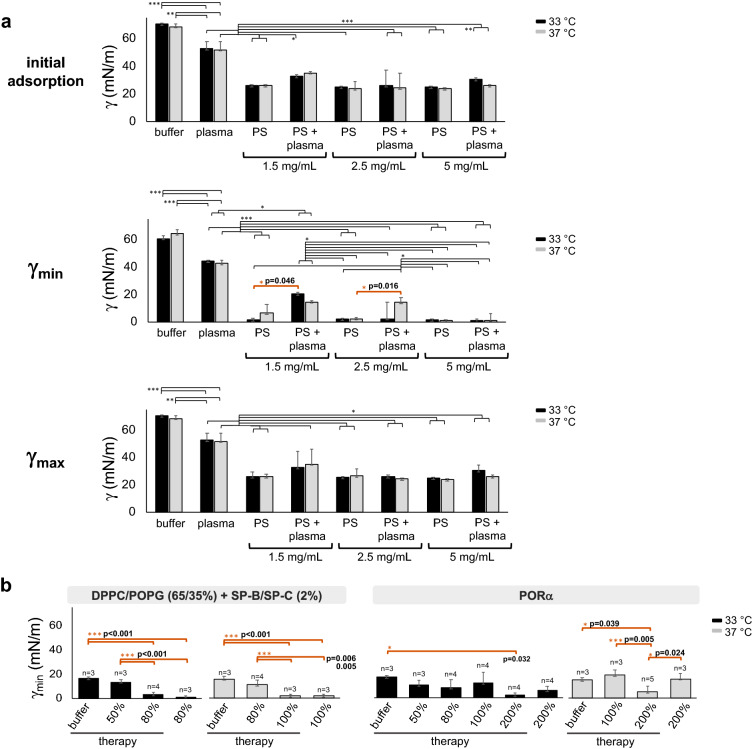
PS inhibition by plasma and restoration at different temperatures. (**a**) CDS experiments of PS are shown at different concentrations at the 2 temperatures (1.5 (n = 4), 2.5 (n = 3) and 5 (n = 3) mg/mL) with and without plasma (68 mg/mL of plasma total proteins (TP)), corresponding to 1.6, 2.6 and 5.2% PLs/plasma TP. Initial adsorption along with minimum and maximum surface tensions upon 10 slow compression-expansion cycles (2 cycles/min, shrinking the interface area by 20%, 0.2 cm^2^/min) are depicted compared to plasma (n = 3) and buffer alone (n = 3). (**b**) Restoration of PS activity after plasma inhibition, with 1.5 mg/mL and 2.5 mg/mL of PS at 33 °C and 37 °C. After inhibiting surfactant, several amounts of further surfactant were supplemented to mimic therapy, using PORα or the lipid-protein mixture [DPPC/POPG (65/35%, w/w) + SP-B/SP-C (2% total protein-to-lipid by weight)]. These supplemented amounts were calculated as the percentage of μg of PLs of therapy with respect to the μg of inhibited PS. As further controls, only buffer was dispensed as a mock therapy for inhibited PS and the restoration therapy was analysed in the presence of plasma alone. Black and light-grey bars represent experiments performed at 33 °C and 37 °C, respectively. Means and standard deviation of at least three replicates are shown. Horizontal lines represent statistical comparisons. Two-way ANOVA test followed by post-hoc test = (a) initial adsorption: temperature (n. s.), PS concentration (p < 0.001, D.F. = 3, F = 139.1) and plasma presence (n. s.); γ_min_: temperature (n. s.), PS concentration (p < 0.001, D.F. = 3, F = 139.1), plasma presence (n. s.), temperature/PS concentration/plasma presence (p < 0.006, D.F. = 3, F = 4.7); γ_max_: temperature (n. s.), PS concentration (p < 0.001, D.F. = 3, F = 124.7) and plasma presence (p < 0.001, D.F. = 1, F = 16.9). Paired t test γ_min_ = 33 °C: PS at 1.5 mg/mL with and without plasma (p = 0.046, D.F. = 3, t =  − 3.3), 37 °C: PS at 2.5 mg/mL with and without plasma (p = 0.016, D.F. = 3, t =  − 5.0). One-way ANOVA test followed by *post-hoc* test = (b) synthetic lipid mixture: 33 °C (p < 0.001, D.F. = 3, F = 101), 37 °C (p < 0.001, D.F. = 3, F = 22.2); PORα: 33 °C (p = 0.044, D.F. = 5, F = 3), 37 °C (p = 0.004, D.F. = 3, F = 8.6). *p < 0.05 and > 0.01, **p ≤ 0.01 and > 0.005, ***p ≤ 0.005. The exact p values of the most relevant *post-hoc* tests are indicated. The corresponding comparison bars and symbols are highlighted in orange color. *PS* purified porcine surfactant, *γ* surface tension, *min* minimum, *max* maximum, *DPPC* dipalmitoylphosphatidylcholine, *POPG* palmitoyloleoylphosphatidylglicerol, *SP-B* surfactant protein B, *SP-*C surfactant protein C, *PORα* poractant α, *n. s.* not significant, *D.F.* degrees of freedom.

The inhibition of PS activity by plasma was tested by CDS upon slow cycling (2 cycles/min) at different surfactant concentrations (1.5, 2.5 and 5 mg/mL), both at 33 and 37 °C (Fig. 4a). Within each condition, no differences between temperatures in initial adsorption and γ_max_ were noticed. Conversely, the dynamic activity (γ_min_) of PS at 1.5 mg/mL was clearly impaired in the presence of plasma (Fig. 4a, second line). Interestingly, at this concentration, PS capability to produce very low surface tension was reduced with and without plasma at 37 °C, but still functional in the absence of plasma at 33 °C (Fig. 4a, second line). Moreover, when PS was tested at 2.5 mg/mL it worked properly in the presence of plasma at 33 °C, but it was inactivated at 37 °C (Fig. 4a, second line).

Restoration experiments (Supplementary Fig. S4) were then performed, maintaining the same amount of plasma, but using different concentrations of PS depending on the experimental temperature: 1.5 mg/mL at 33 °C and 2.5 mg/mL at 37 °C. The restoration activities of two different materials were studied under these conditions: (1) Poractant α (PORα), a widely used therapeutic surfactant, and (2) DPPC/POPG (65/35, w/w) + SP-B/SP-C (2% total protein-to-lipid by weight), the lipid-protein mixture that had shown the best surface-activity (comparable to PS). Several amounts of these materials were used to mimic a therapy, by adding small volumes at increasing concentrations on top of plasma-inhibited surfactant (Supplementary Table S2, Fig. S4) until reaching restoration. These amounts were calculated as the mass percentage of dispensed PLs to PLs in the inhibited surfactant (e.g. 5 μL of PS at 1.5 μg/μL were used at 33 °C, thus 80% of required therapy means 6 μg of therapy). Since the concentration of surfactant material highly influences its surface-active properties^36^, we decided to use the same therapy concentration for both temperatures. This was performed by dispensing higher volumes at 37 °C compared with 33 °C, still maintaining values up to 4% of the constrained drop volume. As a result, the same percentage (with respect to total PLs in inhibited PS) and concentration of therapy were used regardless of the experimental temperature. Moreover, due to the different starting PS concentration (1.5 and 2.5 mg/mL for 33 and 37 °C, respectively), we focused on the therapy percentage necessary to rescue PS activity for data interpretation. The effects of adding buffer alone to the inhibited PS were also analysed as a reference (Fig. 4b).

**Figure 5:**
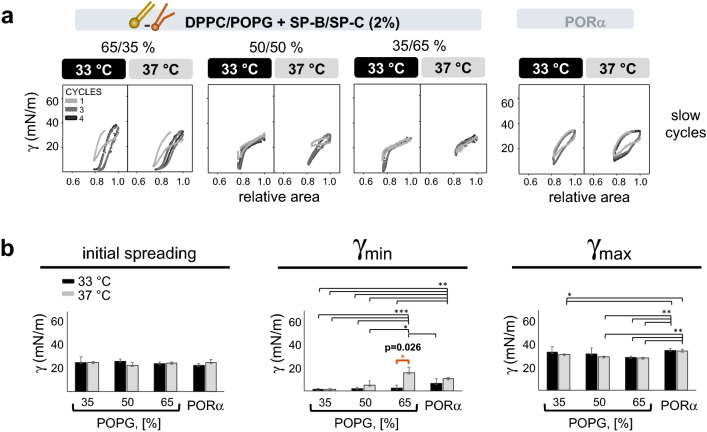
Temperature-mediated resistance to plasma inhibition, depending on DPPC/POPG ratio. About 200 nL at 40 mg/mL of PORα (n = 5 33 °C, n = 3 37 °C) or the lipid-protein mixtures [DPPC/POPG + SP-B/SP-C (2% total protein-to-lipid by weight)] with increasing percentages of POPG (n = 3 for each mixture and temperature), were dispensed at the interface of plasma and subjected to 4 slow cycles in CDS (2 cycles/min, shrinking the interface area by 20%, 0.2 cm^2^/min). (**a**) One representative replicate of γ-∆area isotherms at the 2 temperatures for each tested material. (**b**) On the left, γ values reached after 10 s of material spreading at the plasma interface (initial spreading). In the middle and on the right, γ_min_ and γ_max_ reached during cycling. Compression-expansion cycles are depicted in grey scale. Black and light-grey bars represent experiments performed at 33 °C and 37 °C, respectively. Means and SD of at least three replicates are shown. Horizontal lines represent statistical comparisons. Two-way ANOVA test followed by post-hoc test = initial adsorption: (n. s.); γ_min_: temperature (p < 0.001, D.F. = 1, F = 20.5) and composition (p < 0.001, D.F. = 3, F = 11); γ_max_: temperature (n. s.) and composition (p = 0.003, D.F. = 3, F = 6.8). Paired t test = 65% POPG, γ_min_ at different temperatures: p = 0.027, D.F. = 2, t =  − 6. *p < 0.05 and > 0.01, **p ≤ 0.01 and > 0.005, ***p ≤ 0.005. The exact p values of the most relevant *post-hoc* tests are indicated. The corresponding comparison bars and symbols are highlighted in orange color. *DPPC* dipalmitoylphosphatidylcholine, *POPG* palmitoyloleoylphosphatidylglicerol, *SP-B*: surfactant protein B, *SP-C* surfactant protein C, *PORα* poractant α, *γ* surface tension, *min* minimum, *max* maximum, *n. s.* not significant, *D.F.* degrees of freedom.

Regardless of the therapy used, the amount necessary to restore PS activity was always lower at 33 °C compared with 37 °C. Moreover, the lipid-protein mixture showed better restoration (80–100%), compared to PORα (200%) with a temperature-mediated improvement in the restoration capability. For this material, the restoration dose required was lower at 33 °C than 37 °C (Fig. 4b). This was probably due to the better exclusion of POPG from the interface under compression at 33 °C, leaving enough DPPC to reduce surface tension. To further investigate this hypothesis, three lipid-protein mixtures with decreasing DPPC/POPG ratios were tested with plasma in CDS at 33 and 37 °C and compared to PORα (Fig. 5a,b). Interestingly, the lipid-protein mixtures with DPPC ≥ 50% and POPG ≤ 50%, seem to be more resistant to plasma inhibition than PORα regardless of the experimental temperature. On the contrary, when increasing the amount of POPG to 65%, the activity was inhibited at 37 °C but still functional at 33 °C (Fig. 5b).

## Discussion

We found that TH induces several effects on surfactant properties, compared with the physiological temperature: (1) the capability of PS to drastically reduce surface tension under breathing-like conditions is enhanced in a concentration-dependent manner; (2) surfactant structure and composition at the air–liquid interface re-organize differently under compression (expiration): larger DPPC-enriched condensed domains and lower percentages of less active lipids are detected at 33 °C; (3) PS resistance to plasma inhibition is boosted under cooling; (4) in vitro restoration therapies are more effective at 33 than at 37 °C; (5) the higher the DPPC proportion, the better the restoration performance under TH.

Up to now, no causal therapies are available for ARDS. Although surfactant inhibition is not necessarily the primary pathogenic factor during ARDS^7^, the protein-rich oedema fluid leaking into the alveoli and the local inflammation make its inactivation a secondary and critical step of the syndrome^13,37–39^. This occurs through different biological mechanisms. For instance, several plasma proteins inhibit the proper adsorption of surfactant, creating a steric barrier at the interface (albumin, fibrinogen), fluidifying surfactant membranes (C-reactive protein) or degrading surfactant lipids (lipases, sPLA_2_) and proteins (proteases)^12,13,40^. Here, we observed that the initial adsorption of diluted PS in the presence of plasma was similar at 33 °C and 37 °C (Fig. 4a). However, plasma compounds were better excluded at 33 °C upon interfacial dynamics. Consistently, the γ_min_ was very low regardless of the plasma presence, testing surfactant at 2.5 mg/mL and 33 °C. On the contrary, there was a significant increase in γ_min_ when PS was assayed with plasma at 37 °C (Fig. 4a, second line).

Interestingly, some plasma proteins, such as fibrinogen, can be found into the liquid-expanded phase during lateral compression^41^. Due to the lipid de-mixing, we observed larger condensed (DPPC-enriched) domains at 33 °C and a liquid-expanded phase with larger excluded three-dimensional structures (Fig. 2b, Supplementary Fig. S2). A temperature-dependent appearance of condensed domains during interfacial compression was already described for DPPC and bovine surfactant^42^. Thus, we can speculate that a similar mechanism occurs with plasma compounds. Plasma proteins might be better (at lower pressures) squeezed out from the interface at 33 °C, thus increasing the resistance to plasma inactivation under TH.

Moreover, in both adult and meconial ARDS, high cholesterol and polyunsaturated phosphatidylcholine levels were described as a lung response or due to plasma extravasation and meconium presence^25,43,44^. This may impede surfactant to properly reduce surface tension during expiration. For instance, increasing the amount of cholesterol with respect to DPPC leads to changes in the proper packing of DPPC domains, affecting the capability to rise surface pressures upon lateral compression^45^.

**Figure 6:**
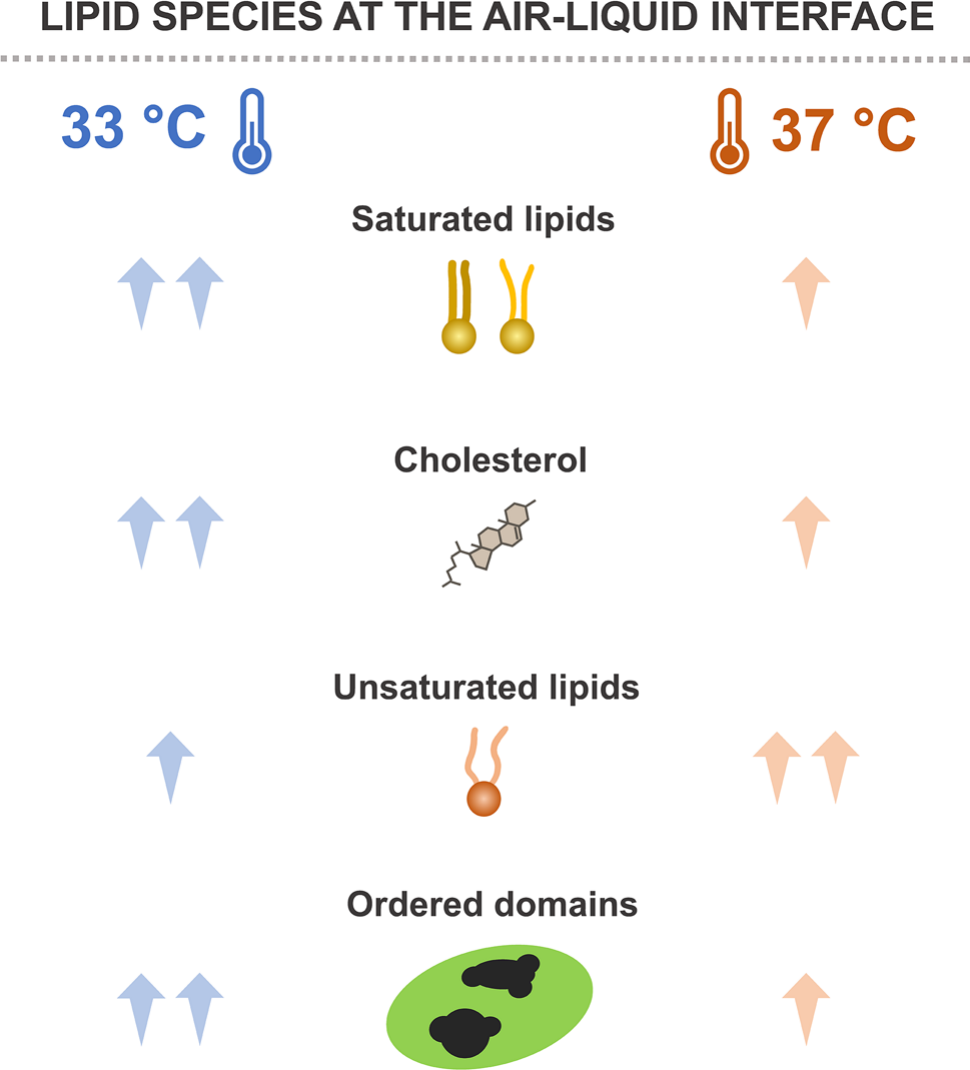
Schematic representation of possible candidates involved in the temperature-mediated improvement of surfactant performance and structural changes at the air–liquid interface. Our findings suggest the presence of higher levels of cholesterol and saturated lipids (mainly DPPC) along with reduced amount of unsaturated lipid species at 33 °C compared with 37 °C. This promotes the formation of ordered condensed domains with bigger size at hypothermia temperature compared with 37 °C.

Interestingly, the surface elasticity of spread DPPC monolayers at high surface pressures seems to lower under increasing temperatures^46^. This reduces the interfacial activity of material, probably due to the quicker relaxation of DPPC monolayers that lose their capability to sustain high surface pressure. In our experiments, an increased percentage of cholesterol and saturated lipids was detected at the air–liquid interface at 33 °C (Fig. 6).

The sterol presence seems to influence the dynamic surface elasticity of DPPC monolayers under compression^47^. However, we still observed a temperature-mediated effect on the lateral organization of DPPC/unsaturated PC domains of PS at the air–liquid interface. This is probably due to the simultaneous increase in both cholesterol and saturated lipids detected at hypothermia temperature. In this regard, we demonstrated formation of larger condensed interfacial domains at 33 °C than at 37 °C during lateral compression (Figs. 2b, 6, Supplementary Fig. S2). Cholesterol may insert preferentially into ordered structures. Thus, a larger DPPC proportion at the interface at 33 °C (Fig. 2d) might cause a higher amount of cholesterol to partition from the surrounding disordered phases. These results may suggest a protective role of TH, reducing the cholesterol-mediated inhibition. This might occur by promoting DPPC de-mixing during compression and indirectly the sterol captures into ordered domains at 33 °C.

**Figure 7:**
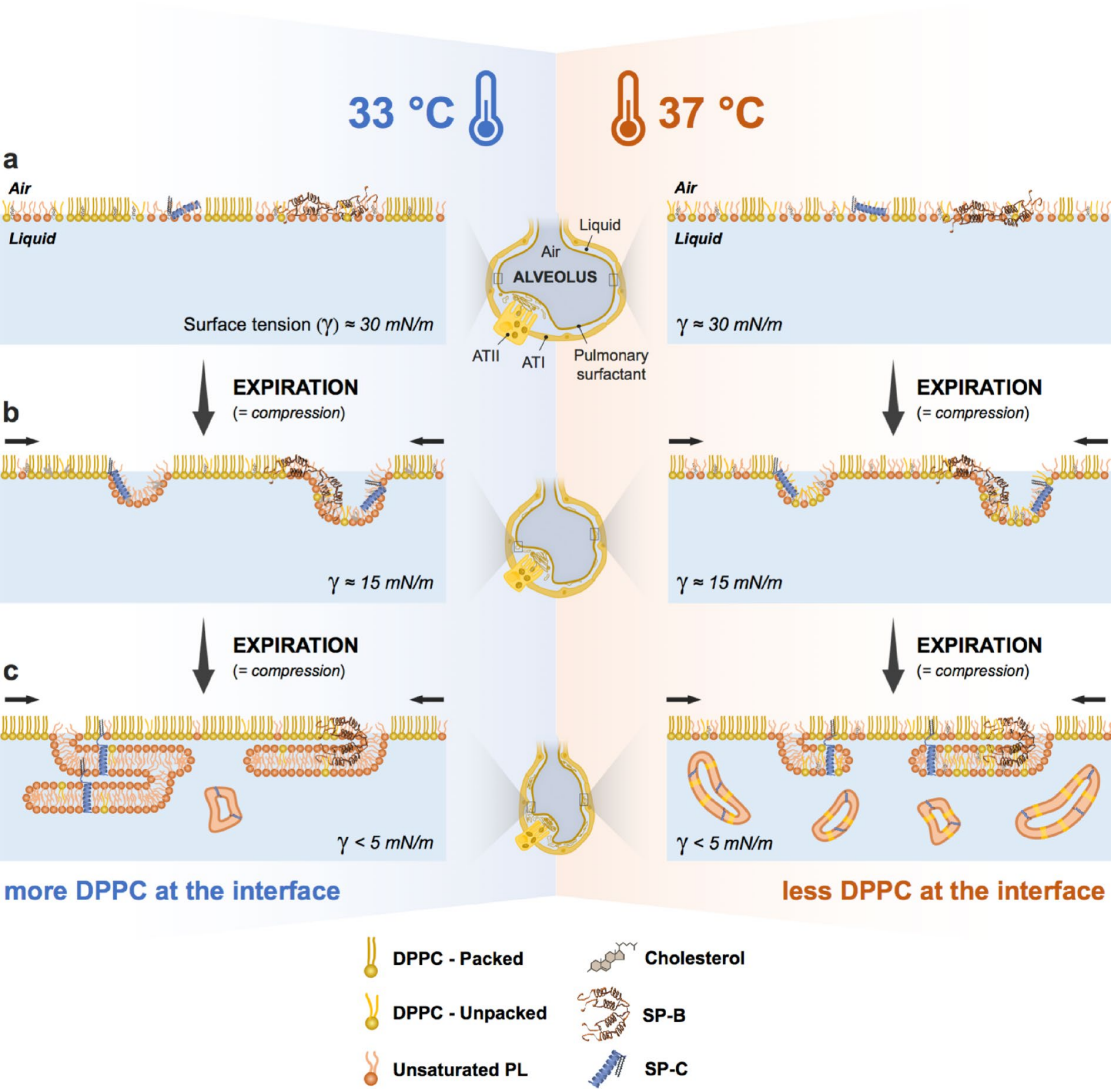
Temperature-mediated compression-driven reorganization of surfactant at the air–liquid interface. (**a**) When surfactant adsorbs at the air–liquid interface, the lateral segregation between packed ordered domains (DPPC enriched) and expanded disordered lipids is less evident at 37 °C due to temperature-facilitated mixing. Larger condensed domains are visible at 33 °C. **(b**) During compression (at expiration), SP-B and SP-C promote surfactant reorganization at the air–liquid interface, facilitating maximal reduction in surface tension. Areas rich in lipids with lower stability at high pressures, and therefore poorer surface-active properties (unsaturated lipids) are laterally and three-dimensional excluded from the air–liquid interface. Due to lipid de-mixing, the condensed ordered/liquid-expanded disordered phase separation is more evident at 33 °C. This leads to compression-driven preferential exclusion of unsaturated lipids. Conversely, a fraction of DPPC is also lost from the interface at 37 °C because of the temperature-dependent partial mixing. (**c**) At high compression rates of the alveolar interface (at the end of expiration), lipids excluded from the interface are organized in three-dimensional structures that remain below. These structures might be more stable and firmly associated to the surface at 33 °C than 37 °C. Indeed, part of those membranes, containing higher DPPC proportions, may detach from the surface at 37 °C. This probably fuels both catabolism and recycling of surfactant lipids under physiological conditions when new surfactant components continuously adsorb and replenish the lost material at the interface. Conversely, the adsorption of surfactant would be compromised by several plasma compounds when they pass from systemic circulation to the alveolar space. This affects the amount of DPPC available to reduce surface tension at 37 °C and suggests a protective role at 33 °C. *SP-B* surfactant protein B, *SP-C* surfactant protein C, *γ* surface tension, *AT-I* alveolar type I cell, *AT-II* alveolar type II cell.

During ARDS, raised levels of sPLA_2_ leads to a significant reduction in both surfactant DPPC^13,37–39^ and PG amounts^21^. At the same time, surfactant proteins decrease^13^ and ROS increase, oxidizing the lipid-protein membranes^12^. As a result, the availability of surface-active material is drastically reduced and surfactant complexes become inactivated. Interestingly, considering our data, our findings suggest that TH could be also beneficial under these impaired surfactant conditions: (1) at lower concentration, as in the ARDS, surfactant performance is better at 33 °C than 37 °C (Fig. 1), (2) surfactant exhibits higher resistance to plasma inhibition under TH (Fig. 4); (3) due to the lower miscibility of lipids at 33 °C, unsaturated lipids (e.g. puPC and DOPC) and plasma components are removed more efficiently from the interface. This occurs with a lower loss of DPPC during the interfacial compression (at expiration) (Figs. 2, 5). This last mechanism (outlined in Fig. 7) fits with the first two effects perfectly. A better performance of surfactant at 33 °C with and without plasma is closely linked to the concentration of tested material. Lower concentrations mean lower DPPC amounts at the air–liquid interface, that are even more reduced under compression at 37 °C.

In asphyxiated neonates, the improvement in surfactant activity under TH seems to be significant after 48–72 h^25^. At this time point, two sPLA_2_ IIA modulators, SP-A and DOPG, might increase and the activity of the enzyme decreases^30^. This may explain the time-dependent effect on surfactant activity under TH, probably related to the higher DPPC amount at the interface.

Finally, we demonstrated an increase in both the endogenous and therapeutic surfactant performance under cooling (Figs. 4, 5). Regardless of the material applied (PORα or lipid-protein mixtures containing different proportions of DPPC and POPG), lower doses were more resistant to plasma inhibition and able to restore PS activity at 33 °C compared with 37 °C. Moreover, the resistance to plasma inactivation was higher at high DPPC ratios regardless of the temperature (Fig. 5). Altogether, these results are promising for primary ARDS patients. In those patients, the maximum beneficial effect of exogenous surfactant should be warranted with the minimum dose.

Our data show a better plasma resistance for the lipid-protein mixture containing ≥ 50% DPPC when compared to PORα at both 33 °C and 37 °C (Fig. 5). Similar observations were recently described for CHF 5633 at physiological temperature, a novel synthetic surfactant that shows promising results in clinical phase II^48,49^. CHF 5633 composition is similar to the lipid-protein mixtures employed in our study: 50/50 DPPC/POPG (w/w%) and 1.8% by mass of SP-B/SP-C analogs^48^. Thus, the mechanism involved in plasma resistance upon dynamic cycles may probably be analogous and particularly enhanced at 33 °C. In this line, the lipid-protein mixture with increased POPG/DPPC ratio showed a reduced activity at 37 °C, but was still functional at 33 °C. We speculate that plasma proteins are excluded from the interface together with POPG during compression, presumably in a process lead by surfactant proteins/peptides. The same mechanism might occur under TH, with less DPPC lost from the interface during compression (Fig. 7). The lower lipid miscibility at 33 °C may contribute to reduce DPPC mixing into the liquid-expanded phase and its interfacial exclusion under compression. This would explain the good performance of the material at low DPPC ratio (35%) (Fig. 5).

We acknowledge some study limitations. PS undergoes a temperature-mediated collapse under compression in the Langmuir–Blodgett balance^50^. This prevents analysing surfactant interfacial structure and composition at surface pressures higher than 40 mN/m. In addition, we could not perform a lipidomic analysis after compression-expansion cycles since these experiments last around 30 min and the lipids at the interface may oxidize during this period. Also, experiments using lipid-protein mixtures containing only SP-C could not be carried out in the CBS, because the presence of SP-B is indeed essential for the adsorption and re-adsorption of a significant proportion of lipids at the bubble interface^51^. As previously described, surfactant is resistant to plasma inactivation under quick cycles in the CDS^52^. For that reason, the use of slow cycles was necessary to facilitate the competition of plasma proteins for the air–liquid interface. Finally, we cannot exclude that the lower interfacial activity at 37 °C, especially for the mixture containing high percentages of POPG or DOPC, was influenced by a higher lipid oxidation at increased temperatures^53^.

Altogether, we demonstrated that TH influences surfactant structure and composition at the air–liquid interface, increasing the interfacial proportion of DPPC upon compression. This leads to improved lung and therapeutic surfactant activity both under physiological and inhibitory conditions. Our evidences should be added to the multiple effects of TH on human lungs.

## Methods

### Materials

PS and purified SP-B and C were obtained from porcine bronchoalveolar lavage (BAL) as previously reported^15^ (Supplementary Fig. S5). The experimental procedures are described in the supplementary information. Porcine plasma was obtained by centrifuging (500×*g* for 10 min at 4 °C) and pooling blood from 6 pigs with different sexes coming from the same slaughterhouse. Similarly, PS was purified from the BAL pool obtained from the 6 pig lungs. Animals used to recover these materials were healthy and subjected to Vet control according to local regulations (Spanish hygiene rules legislation, law articles 83, 85 and 178). Pigs were sacrificed for food and not for the sole purpose of the study.

PS and synthetic PLs concentrations were determined by phosphorus mineralization^54^. Plasma total protein (TP) concentration was measured with the Lowry protein assay^55^.

PORα is a widely available surfactant obtained by minced porcine lung and considered to be the most efficacious in neonatal critical care, according to evidence based data and European guidelines^56,57^. It was purchased from Chiesi Farmaceutici S.p.A. (Parma, Italy), lyophilized and stored at − 80 °C until use. DPPC, POPG, POPC and DOPC were purchased from Avanti Polar Lipids, Inc. (Alabaster, Alabama, USA). BODIPY-PC was purchased from Molecular Probes (Life Technologies, Carlsbad, California, USA). The same buffer solution containing 5 mM Tris and 150 mM NaCl at pH 7.4 was used for every experiment.

### Biophysical activity and lateral structure of surfactant films

The biophysical activity of both PS and lipid-protein surfactant suspensions was tested at limiting concentrations (1.5, 2, 5 and 8 mg/mL) under slow (2 cycles/min) and quick breathing-like (20 cycles/min) compression-expansion cycling by CDS^58^ and CBS^51^ (Supplementary Fig. S6). Surfactant capability to reduce surface tension was not inhibited during quick cycles in the CDS after bulk replacement with serum^52^. Thus, both plasma inhibition and therapy restoration experiments were performed under slow cycles (Supplementary Fig. S6, Table S2). A Langmuir–Blodgett surface balance was used to study PS lateral structure (Supplementary Fig. S8) before and after subjecting material (around 30 μg at 5 mg/mL) at the interface to 10 compression-expansion cycles (65 cm^2^/min).

All methods were applied keeping the target temperature (33 °C or 37 °C) constant along the experiments. Details about protocols and techniques are described in supplementary information.

### Composition of interfacial films and thermotropic properties

The temperature-dependent changes in lipid composition of PS films under compression at the air–liquid interface were analysed in a Langmuir–Blodgett balance (Supplementary Fig. S8). Briefly, PS (around 50 μg, 5 mg/mL), pre-heated to 33 °C or 37 °C, was deposited drop by drop at the air–liquid interface of the trough filled with buffer at the same temperature. After 10 min of equilibration (≈ 25 mN/m surface pressure), surfactant films were compressed, reducing interfacial area at a velocity of 25 cm^2^/min and simultaneously transferred onto a glass slide. Immediately after drying, the glass slide was rinsed with chloroform/methanol (2:1 v/v) to collect lipids from the film. Five experiments per condition were pooled in a single sample for lipidomic analysis by liquid chromatography-high resolution mass spectrometry (LC-HRMS)^43^. Each lipid class was expressed as molar % of the total PLs.

DSC was performed on both PS and lipid/protein suspensions, testing materials at 3 mg/mL as described earlier^48^ and in supplementary information.

### Statistics

Data were expressed as mean (standard deviation). Results comparisons were conducted using One-Way ANOVA followed by *post-hoc* Tukey’s test or Two-Way ANOVA followed by post-hoc paired and unpaired T-test when appropriate. Details are described in figure captions. Correlation analysis was performed using Spearman (ρ) coefficient. Analyses were carried out with Sigma Plot (v. 11, Systat Software, San Jose, USA) and IBM SPSS Statistics 25 (v.25, IBM Corp., Armonk, NY, USA). p < 0.05 was considered to be significant.

## Supplementary Information

Supplementary Information.
