# Author Correction: Molecular and biophysical mechanisms behind the enhancement of lung surfactant function during controlled therapeutic hypothermia

**DOI:** 10.1038/s41598-021-89532-6

**Published:** 2021-05-05

**Authors:** C. Autilio, M. Echaide, A. Cruz, C. García-Mouton, A. Hidalgo, E. Da Silva, D. De Luca, Jorid B. Sørli, J. Pérez-Gil

**Affiliations:** 1 Department of Biochemistry and Molecular Biology and Research Institute “Hospital 12 de Octubre (imas12), Faculty of Biology, Complutense University, Jose Antonio Novais 12, Madrid, Spain; 2 National Research Centre for the Working Environment, Copenhagen, Denmark; 3 Department of Environmental Engineering, Technical University of Denmark, Kgs. Lyngby, Denmark; 4 Division of Pediatrics and Neonatal Critical Care, “A.Béclère” Medical Center, Paris Saclay University Hospitals, APHP, Paris, France; 5 Physiopathology and Therapeutic Innovation Unit-INSERM U999, South Paris-Saclay University, Paris, France

Correction to: *Scientific Reports* https://doi.org/10.1038/s41598-020-79025-3, published online 12 January 2021

The original version of this Article contained errors in the spelling of the authors C. García-Mouton, Jorid B. Sørli and J. Pérez-Gil which were incorrectly given as C. Mouton, B. Sørli Jorid and J. Perez-Gil respectively.

These errors have now been corrected in the PDF and HTML versions of the Article, and in the accompanying Supplementary Information file.

