# Supplementary material for: Molecular and biophysical mechanisms behind the enhancement of lung surfactant function during controlled therapeutic hypothermia
Source: Sci Rep. 2021 Jan 12;11:728. doi: 10.1038/s41598-020-79025-3 (PMC7804441; doi:10.1038/s41598-020-79025-3)

**Molecular and biophysical mechanisms behind the enhancement of lung surfactant function during controlled therapeutic hypothermia**

Autilio C^1^, Echaide M^1^, Cruz A^1^, García-Mouton C^1^, Hidalgo A^1^, Da Silva E^2,3^, De Luca D^4,5^, Sorli JB^2^ and Pérez-Gil J*^1^

**Online supplementary information**

*Porcine surfactant purification*

Porcine bronchoalveolar lavages (BALs) were obtained by washing porcine lungs with buffer (5 mM Tris and 150 mM NaCl at pH 7.4) (**Supplementary** **Fig S5a**). Subsequently, BALs were centrifuged (1000 g for 10’ at 4 °C) to remove cells and debris and the obtained supernatant was ultra-centrifuged at 100,000 g for 1h (70 Ti fixed-angle rotor; Beckman Coulter, Brea, CA) at 4 °C. The resulting pellets were resuspended in a sodium bromide solution (16% NaBr and 0.9% NaCl) with a Potter-Elvehjem homogenizer. These homogenized pellets were loaded onto a sodium bromide gradient centrifugation to remove potential blood contaminants, as previously described^1^. In detail, two saline solutions were carefully added to 1 volume of resuspended pellets (16% NaBr/ 0.9% NaCl), following this order: 1.5 volume of (13% NaBr/ 0.9% NaCl) and 0.6 volume of 0.9% NaCl. The suspensions were then ultra-centrifuged at 120,000 g for 2h at 4 °C (SW40 Ti swinging-bucket rotor; Beckman Coulter). After centrifugation, purified porcine surfactant (PS) was obtained as a disc between the 13% NaBr/ 0.9% NaCl solution and the 0.9% NaCl cushion layers. This surfactant disc was then collected, resuspended in 0.9% NaCl and stored in aliquots at -80 °C. PS phospholipid concentration was assayed by phosphorus mineralization^2^, resulting in around 80 mg/mL.

*Purification of porcine SP-B and SP-C*

Porcine hydrophobic surfactant proteins SP-B and SP-C were purified by two sequential size-molecular exclusion chromatography (MEC) steps, as described earlier^3^, using the organic extract of PS (**Supplementary** **Fig S5a**). In detail, milliQ water, pure methanol and chloroform (Sigma-Aldrich, St. Louis, MO, USA) were added to PS in the following volume proportions: 1:1:2:1 (PS:Water:Methanol:Chloroform). After mixing thoroughly for 30’’, the obtained solution was incubated at 40 °C for 30’ to allow for the water-soluble protein flocculation. Subsequently, one more volume of chloroform and water were added to the sample, before mixing thoroughly for 30’’. The obtained biphasic solution was centrifuged for 5’ at 600 g at 4 °C. The organic fraction formed at the bottom of the tube, namely PS organic extract (OE), was collected. Other 2 volumes of chloroform were added to the remained aqueous fraction, repeating the centrifugation step. This last process was performed twice to yield full hydrophobic material recovery. OE was stored at -20 °C. Its phospholipid concentration was assayed by phosphorus mineralization^2^ and resulted in around 20 mg/mL.

After OE concentration in a rotary evaporator, OE hydrophobic constituents were eluted by MEC using a gel filtration resin (Sephadex LH-20, GE Healthcare; Little Chalfont, UK), with Chloroform:Methanol (2:1, v/v) as the eluent in a 1000mm-SR25 column (Ge Healthcare, Little Chalfont, UK). The LH-20 resin allows for the separation by gravity of three OE fractions according to their size and structure: hydrophobic surfactant proteins (SP-B/SPC: 42% SP-B and 58% SP-C^4^), phospholipids and neutral lipids. Subsequently, a fraction of delipidised SP-B/SP-C was used to separate the 2 surfactant proteins by a gel filtration resin with larger pore diameter (Sephadex LH-60, GE Healthcare; Little Chalfont, UK) in Chloroform:Methanol (1:1, v/v) acidified with 0.05% HCl (0.1N, to reduce hydrophobic interactions between the proteins and the resin). The LH-60 resin allows for the separation by gravity of hydrophobic proteins according to their size, the bigger (SP-B) eluting before than the smaller (SP-C).

The protein concentration of SP-B/C and SP-B fractions was quantified by amino acid analysis in a High-Performance Liquid Ion-Exchange Chromatographer. In detail, a volume of protein fractions was dried under a nitrogen flow and subjected to acidic hydrolysis (HCl 6N 0.1% phenol w/v containing a known amount of *nor*-leucine as internal standard) for 24h at 100 °C under vacuum. After hydrolysis, HCl was evaporated under a nitrogen flow and dried tubes were subjected to two milliQ washing and evaporation steps (SpeedVac Concentrator System, Thermo Fisher Scientific; Waltham, MS). Hydrolysed samples were resuspended in citrate buffer for injection into the analyzer. Knowing the amino acid sequence of each protein, the amino acids present in both SP-B and SP-C were used to quantify the concentration of whole SP-B/C fraction (LH-20) and the purified SP-B fraction (LH-60). Each concentration was corrected considering the internal standard. SP-B/C concentration resulted in around 200 μg/mL, whereas SP-B concentration was in the range of 30-40 μg/mL.

Around 10 μL of each purified surfactant fraction was used to load a polyacrylamide Gel Electrophoresis (PAGE, 16% polyacrylamide) followed by a Western blot analysis as previously described^5,6^. This allowed to verify the presence of both SP-B and C in the LH-20 fraction and excluded a cross-contamination by SP-C in the SP-B LH-60 fraction (**Supplementary Fig S5b**). In detail, electrophoresis Laemmli buffer (2% SDS, 62.5 mM Tris pH 6.8, 10% glycerol, 0.03% bromophenol blue) containing 4% β-mercapto-ethanol was added to the dried protein fractions. Samples were incubated 15’ at 90°C and loaded onto the gel. The latter was run around 1h and proteins were transferred onto PVDF membranes with a semi-dry system (1h at 4°C) and blocked in PBS-T (100mM Na_2_HPO_4_/KH_2_PO_4_-1% Tween) with 5% skimmed milk at room temperature for 2h. Membranes were then incubated overnight with the primary antibody [1:5000, anti-mature SP-B or anti-mature SP-C from Seven Hills, (Cincinnati, OH-USA)] in PBS-T 5% milk at 4°C, washed 5 times in PBS-T and incubated with the secondary antibody [1:5000, antirabbit (P0217) by Dako (Agilent, Santa Clara, CA-USA)] for 1h at room temperature. Membranes were then developed (1’ of exposition) using a commercial ECL system (Millipore, Burlington, MA-USA).

*Samples-materials handling*

Phospholipids DPPC, POPC, POPG and DOPC (Avanti Polar Lipids, Inc., USA), bought as dry powders, were weighed and resuspended in Chloroform:Methanol (2:1, v/v) before quantifying their concentration by phosphorus mineralization^2^. Subsequently, different volumes of lipids and surfactant proteins were mixed as organic solutions according to the required proportions. These mixtures were dried under a nitrogen flow, resuspended in buffer to reach a final concentration of 8 mg/mL (5 mM Tris and 150 mM NaCl at pH 7.4) and incubated 1h at 45 °C by intermittent shaking (every 10’, 1400 rpm) to reconstitute multilamellar suspensions. The samples were than chilled to 37 or 33 °C before testing their activity.

PS was diluted in buffer to reach the experimental concentrations. PORα (from Chiesi farmaceutici, Parma) was lyophilized and resuspended in buffer at the time of the experiments, using different volumes to achieve the required final concentrations, and incubated 1h at 37 °C before testing. To perform the epifluorescence experiments, PS was doped with BODIPY-PC (Thermo Fisher Scientific, Massachusetts, USA) at 1% mol/mol, incubating surfactant and probe for 1h at 37 °C with intermittent shaking (every 10’, 1400 rpm). BODIPY-PC stock was resuspended in DMSO (Dimethyl sulfoxide) at 1 mg/mL. Plasma aliquots (68 mg/mL of TP) were used immediately after thawing.

*Surfactant dynamic properties*

Adsorption and dynamic properties of lipid-protein mixtures and PS under breathing-like conditions were tested by Captive Bubble Surfactometer (CBS) and Constrained Drop Surfactometry (CSD). An illustrative representation of the two surfactometers is shown in **Supplementary Figure S6**. These devices recreate an alveolus *in vitro* as an air-liquid interface subjected to continuous breathing-like compression-expansion cycles at controlled temperatures. CBS is characterized by an air bubble, resting on an agarose roof, and enclosed in a liquid chamber (**Supplementary Fig. S6a)**. CDS consists of a buffer drop enclosed in an air chamber and connected to a syringe (**Supplementary Fig S6b)**. Both the bubble and the drop are continuously recorded during experiments by a video camera. Temperature is kept constant by a heater in CBS and both a heater and a Peltier module in CDS. Changes in temperatures are checked overtime using temperature probes to maintain a maximum temperature variation of around ±1 °C.

Samples can be injected below or onto the bubble/drop surfaces to study adsorption and spreading surfactant properties, respectively. Once the material is adsorbed or spread, compression-expansion cycles are performed moving a piston up and down above the chamber in CBS or moving the drop volume in CDS (**Supplementary Fig. S6**).

The presence of a surfactant material that is well organized at the air-liquid interface and able to reduce surface tension leads to changes in the shape of both the bubble and the drop. In fact, surfactant coats the liquid surface, decreasing the proximity between liquid molecules and air. This leads to variations in the surface geometry, resulting in a disc-like shape of both the bubble and the drop under low γ values (< 5 mN/m, **Supplementary Fig. S6**). In both devices, these changes in shape can be monitored overtime and transformed into γ variations by Axisymmetric drop shape analysis (ADSA) software^7^ in CDS and an analogous software designed by Schoel^8^ in CBS.

CBS experiments were performed by injecting around 300 nL of material (PS or lipid-protein mixtures at 1.5 or 8 mg/mL) onto the bubble and waiting 5’ for surfactant adsorption. The chamber was then sealed and the bubble was expanded, increasing its volume by ≈25%. After 5 minutes of this post-expansion, a slow compression-expansion cycle was performed. In detail, the bubble was compressed and expanded once during 2 minutes, changing its volume by ≈25%. Finally, the bubble was subjected to 20 quick cycles, maintaining the same variation of the bubble volume (20 cycles/min).

As for CDS, PS (5 μl at 1.5, 2.5 and 5 mg/mL) was dispensed on top of the pedestal and kept down until reaching the buffer inside the pedestal. A buffer drop was then added on top (7μl), waiting 10’’ (initial adsorption) before performing 10 slow compression-expansion cycles (2 cycles/min, shrinking the interface area by 20 %) (**Supplementary Fig. S6b**).

For lipid-protein mixtures, materials (40 mg/mL) were dispensed directly on top of the plasma drop (previously subjected to 6 slow compression-expansion cycles), and subjected to further 4 slow cycles. Restoration experiments are described in detail in **Supplementary Figure S7**. The concentrations and volumes of rescues (“therapies”) were summarized in **Supplementary Table S2**. The linearity of the 1 μL syringe used to leave therapy at the air-liquid interface was also studied and shown in **Supplementary Figure S7**. To do so, several volumes of the protein-lipid mixture used for the restoration experiments (at ≈ 25 mg/mL of phosphatidylcholine (PC)), were dispensed in triplicate at the air-liquid interface of a buffer drop (10 μL). The resulting PC amounts were tested by an enzymatic method (Spinreact, Girona, Spain) and compared to the theoretical values. There was a strong correlation (ρ=1) between theoretical and obtained values, suggesting very small errors in dispensing material.

*Langmuir Blodgett Balance*

A Langmuir-Blodgett balance (total area= 184 cm^2^, NIMA Technology, Inc., Coventry, UK) was used to study PS interfacial activity and lateral structure (**Supplementary Fig. S8**) before and after subjecting material at the interface to 10 compression-expansion cycles (65 cm^2^/min). This device is characterized by a Teflon trough filled with buffer on top of a Peltier module to heat the liquid. Buffer subphase was maintained wormer than the interface to reach the target temperature at the air-liquid interface. The latter was continuously recorded by a temperature probe resting at the interface and away from any possible contact with

the walls of the trough. The maximum temperature variation was around ±1°C. Compression-expansion cycles were performed by moving a Teflon ribbon barrier, shrinking the interface area by ≈65 %. This continuous Teflon ribbon barrier encloses the interface and avoids the film leakage during compression^9^.

Surfactant material is deposited dropwise at the air-liquid interface and the reduction in surface tension is recorded overtime as increase in surface pressure against a paper plate sensor. The interfacial compression is started 10’ after surfactant deposition. The sample at the air-liquid interface can eventually be transferred onto a glass slide, moving up the glass (5 mm/min) coupled with the interface compression (25 cm^2^/min), as illustrated by the red arrows in **Supplementary Figure S8**. As for epifluorescence experiments, around 30 μg of PS at 5 mg/mL previously doped with BODIPY-PC (1% mol/mol) was dispensed at the interface and transferred onto the glass slide before and after 10 cycles (65 cm^2^/min), performing experiments in darkness. Lateral segregated domain structures of PS were observed by epifluorescence microscopy (Olympus BX-60 microscope with an Olympus DP71 camera). Two replicates per condition were carried out and 4 images for each tested surface pressure were acquired per replicate. For each image, histogram stretching has been performed to enhance contrast without deleting pixel data by Adobe Photoshop CS4. All the original and modified images used for the analysis are available below.

As regards lipidomic analysis, around 50 μg of PS at 5 mg/mL was dispensed at the interface, before transferring material (25 cm^2^/min). Lipids at the interface were rinsed with chloroform:methanol (2:1, v/v) and dried by a nitrogen flow (around 5’). Five replicates per condition were pooled and analyzed by LC-HRMS. Each lipid class was expressed as molar % of the total phospholipids (PLs).

*Differential Scanning Calorimetry*

Differential Scanning Calorimetry experiments were performed as follows. Each lipid-protein mixture resuspended in buffer was assayed at 3 mg/mL. PS was diluted to reach the same concentration before testing. The sample and the reference material (buffer) were degassed prior to being inserted in the two microcalorimeter pans of a VP-DSC microcalorimeter (No. MC-2, MicroCal, Amherst, MA, USA). Samples were consecutively heated to 60 °C and cooled to 2 °C (30 °C/h) for 10 cycles of temperature scanning. The same heat was applied to the sample and the reference cells. Data from the 10^th^ cycle were analyzed by the software Origin (Origin Labs, Northampton, MA, USA). Thermograms and melting temperatures for each condition are shown in **Supplementary Figure S3**. As described in literature^10^, the presence of both hydrophobic proteins (light-grey plot) increases the melting temperature in each condition, shifting the thermogram to right in the graphs.

**References**

1 Taeusch, H. W., De La Serna, J. B., Perez-Gil, J., Alonso, C. & Zasadzinski, J. A. Inactivation of pulmonary surfactant due to serum-inhibited adsorption and reversal by hydrophilic polymers: experimental. *Biophysical journal* **89**, 1769-1779 (2005).

2 Rouser, G., Siakotos, A. & Fleischer, S. Quantitative analysis of phospholipids by thin‐layer chromatography and phosphorus analysis of spots. *Lipids* **1**, 85-86 (1966).

3 Perez-Gil, J., Cruz, A. & Casals, C. Solubility of hydrophobic surfactant proteins in organic solvent/water mixtures. Structural studies on SP-B and SP-C in aqueous organic solvents and lipids. *Biochim Biophys Acta* **1168**, 261-270, doi:10.1016/0005-2760(93)90181-8 (1993).

4 Schürch, D., Ospina, O. L., Cruz, A. & Pérez-Gil, J. Combined and independent action of proteins SP-B and SP-C in the surface behavior and mechanical stability of pulmonary surfactant films. *Biophysical journal* **99**, 3290-3299 (2010).

5 Autilio, C. *et al.* Surfactant Injury in the Early Phase of Severe Meconium Aspiration Syndrome. *Am J Respir Cell Mol Biol* **63**, 327-337, doi:10.1165/rcmb.2019-0413OC (2020).

6 Lopez-Rodriguez, E., Roldan, N., Garcia-Alvarez, B. & Perez-Gil, J. Protein and lipid fingerprinting of native-like membrane complexes by combining TLC and protein electrophoresis. *J Lipid Res* **60**, 430-435, doi:10.1194/jlr.D090639 (2019).

7 Yu, L. M. *et al.* Constrained sessile drop as a new configuration to measure low surface tension in lung surfactant systems. *Journal of applied physiology* **97**, 704-715 (2004).

8 Schoel, W. M., Schurch, S. & Goerke, J. The captive bubble method for the evaluation of pulmonary surfactant: surface tension, area, and volume calculations. *Biochim Biophys Acta* **1200**, 281-290, doi:10.1016/0304-4165(94)90169-4 (1994).

9 Cruz, A. & Perez-Gil, J. Langmuir films to determine lateral surface pressure on lipid segregation. *Methods Mol Biol* **400**, 439-457, doi:10.1007/978-1-59745-519-0_29 (2007).

10 Shiffer, K. *et al.* Lung surfactant proteins, SP-B and SP-C, alter the thermodynamic properties of phospholipid membranes: a differential calorimetry study. *Biochemistry* **32**, 590-597, doi:10.1021/bi00053a026 (1993).

**Supplementary Figure S1. Changes in PS activity depending on temperature and concentration under slow cycles.**

**a)** Minimum and maximum surface tensions reached during cycles 1, 5 and 10, testing surfactant at 2.5 mg/mL (n= 3). **b)** Minimum and maximum surface tensions reached during cycles 1, 5 and 10, testing surfactant at 5 mg/mL (n= 3).

Black and light-grey bars represent experiments performed at 33 °C and 37 °C, respectively. Mean and SD of three replicates are shown. Horizontal lines represent statistical comparisons. Two-way ANOVA test followed by *post-hoc* test= a) γ_min_: temperature (n. s.) and cycles (p< 0.001, D.F.= 2, F= 159), γ_max_: n. s.; b) γ_min_: temperature (n. s.) and cycles (p< 0.001, D.F.= 2, F= 60.0), γ_max_: temperature (n. s.) and cycles (p= 0.014, D.F.= 2, F= 6.25). * p< 0.05 and > 0.01, *** p≤ 0.005. The exact p values of the most relevant *post-hoc* tests are indicated. The corresponding comparison bars and symbols are highlighted in orange color. Abbreviations: γ: surface tension; min: minimum; max: maximum; D.F.: degrees of freedom; n. s.: not significant.

**Supplementary Figure S2. Temperature-dependent changes in surfactant lateral structure under compression in a Langmuir-Blodgett through.**

**a)** One representative replicate (n= 2) of Π-Δarea isotherms before and during 10 slow compression-expansion cycles of the interface (0.3 cycles/min, ≈65 cm^2^/min, shrinking the interface area by ≈65 %) at 33 °C or 37 °C. Around 30 μg (at 5 mg/mL) of PS previously doped with BODIPY-PC (1% mol/mol) were dispensed at the interface. Cycles 1, 5 and 10 are shown. **b)** Representative images and statistical analysis of condensed and expanded regions observed at 33 °C and 37 °C, transferring the interface upon compression after 10 compression-expansion cycles. On the left, temperature-dependent changes in the count of black condensed domains with different diameters (< 30 μm, 30-150 μm, > 150 μm) at several surface pressures (18, 25, 30 mN/m). Due to the heterogenous and irregular shapes, the longer diameter for each condensed domain was considered for the analysis. An orange dotted line was shown as an example. On the right, temperature-dependent changes in the count of bright green spots per field at several surface pressures (10, 18, 25 and 30 mN/m). White scale bars, 100 μm. At high surface pressures (> 25 mN/m), the size of black condensed domains increases at 33 °C compared with 37 °C along with the number of bright green spots (> 18 mN/m).

Black and light-grey colors represent experiments performed at 33° C and 37 °C, respectively. Mean and SD of 8 images per condition are shown. For each image, histogram stretching has been performed to enhance contrast without deleting pixel data. Black horizontal lines represent statistical comparisons.

Two-way ANOVA test followed by *post-hoc* test: b) < 30μm: temperature (p= 0.016, D.F.= 1, F= 6.3) and surface pressure (n. s.), 30-150 μm: temperature (n. s.) and surface pressure (p= 0.004, D.F.= 2, F= 6.3), > 150 μm: temperature (p< 0.001, D.F.= 1, F= 19.5) and surface pressure (n. s.); fluid brilliant green spots: temperature (p< 0.001, D.F.= 3, F= 85.7) and surface pressure (p= 0.011, D.F.= 1, F= 4.1). * p< 0.05 and > 0.01, ** p≤ 0.01 and > 0.005, *** p≤ 0.005. The exact p values of the most relevant *post-hoc* tests are indicated. The corresponding comparison bars and symbols are highlighted in orange color.

Abbreviations: Π: surface pressure; D.F.: degrees of freedom; n. s.: not significant.

**Supplementary Figure S3. Differential scanning calorimetry of PS and lipid-protein mixtures used in CBS experiments.**

**a)** One representative thermogram (n= 3) for PS, lipid mixtures alone, lipid mixtures with SP-B (1% w/w) and lipid mixtures with SP-B/SPC (2% w/w). The presence of both hydrophobic proteins (light-grey plot) increases the melting temperature in each condition, shifting the thermogram to right in the graphs. The presence of SP-B alone increases the melting temperature for the lipid-protein mixtures containing DPPC and POPG. As for this mixture, the hydrophobic proteins also increase the cooperativity. **b)** Summary table of the different melting temperatures. The melting temperatures of PS, [DPPC/POPG + SP-B] and [DPPC/POPG + SP-B/SP-C] mixtures are similar and close to the temperature used during moderate hypothermia (33 °C).

Abbreviations: Cp: Specific heat capacity; Tm: melting temperature; DPPC: dipalmitoylphosphatidylcholine; POPG: palmitoyloleoylphosphatidylglicerol; POPC: palmitoyloleoylphosphatidylcholine; DOPC: dioleoylphosphatidylcholine; PS: porcine surfactant; SP-B: surfactant protein B; SP-C: surfactant protein C.

**Supplementary Figure S4. Technical protocol of a CDS experiment of PS inhibition and restoration.**

CDS was maintained at the target temperatures (33 or 37 °C) by using a thermostatic chamber and temperature was continuously recorded by 2 internal probes: the first one close to the syringe containing the buffer and the second one close to the drop.

The restoration experiment was carried out in around 6’ with the following steps:

1. 5 μL of PS at different concentrations was left on top of the pedestal and taken down slowly into the pedestal, which was filled with buffer.
2. Subsequently, 7 μL of plasma (or buffer, for the control) were dispensed on top of the pedestal. Plasma was dispensed immediately after thawing. After 10’’, materials at the interface were subjected to slow expansion-compression cycles (2 cycles/min, shrinking the interface area by 20 %, 0.2 cm^2^/min). As for the restoration experiments, the syringe movement was stopped at the end of the seventh expansion.
3. At this point, therapeutical treatment was applied at the air-liquid interface of the drop, touching once the drop. A syringe of 1 μL was used, dispensing small volumes (150-600 nL). The same slow cycles were started again for another 5 cycles.

Abbreviations: PS: purified porcine surfactant; μ: surface tension; min: minimum; max: maximum.

**Supplementary Figure S5. Schematic protocol for PS purification and isolation of SP-B/SP-C protein fraction and SP-B.**

**a)** Scheme of the procedure applied to isolate porcine hydrophobic proteins from BALs of porcine lungs, using sephadex LH-20 (SP-B/C), followed by LH60 columns (SP-B). **b)** Western blot analysis, using anti-SP-B and anti-SP-C antibodies to test the presence of the two proteins and their cross-contaminations into the eluted fractions. 10 μL of sample was loaded onto each lane of the polyacrylamide gel. This volume corresponded to around 2 μg of SP-B/C and 0.3-0.5 μg of SP-B. Moreover, several amounts of SP-B purified from porcine lung tissues were also loaded as internal controls (L-SP-B).

Abbreviations: BAL: bronchoalveolar lavage; PS: purified porcine surfactant; MEC: molecular exclusion chromatography; SP-B: surfactant protein B; SP-C: surfactant protein C; LH-20 and LH-60: sephadex column; PLs: phospholipids; NLs: neutral lipids; L-SP-B: SP-B purified from porcine lung tissues; α: anti.

**Supplementary Figure S6. Surfactometers used to study biophysical activity of material under slow and quick compression-expansion cycling.**

**a)** Schematic representation of CBS and experimental protocol for initial adsorption and dynamic quick compression-expansion cycles (20 cycles/min). Temperature was kept constant along the experiment and checked by a probe overtime. The maximum temperature variation was around ±1°C.

**b)** Schematic representation of CDS and experimental protocol for initial adsorption and dynamic slow compression-expansion cycles (2 cycles/min). Temperature was kept constant along the experiment and checked by two probes overtime: the first one close to the syringe containing the buffer and the second one close to the drop. The maximum temperature variation was around ±1°C.

Abbreviations: PS: purified porcine surfactant; γ: surface tension.

**Supplementary Figure S7**. **Dispensing linearity of a 1μL syringe, leaving material at the interface of a buffer drop (10 μL) at room temperature.** The protein-lipid mixture containing DPPC/POPG (65/35, w/w%) and SP-B/C (2% of total w) at ≈25 mg/mL was used for the experiments. On the left, comparison of theoretical values of PC amount and obtained values determined by an enzymatic assay. Results for different volumes (100, 150, 300 and 600 nL) of material were analyzed. On the right, correlation between theoretical and obtained values, using a Spearman (rho) coefficients. Results show a good linearity and a strong correlation (ρ=1), suggesting very small errors in dispensing the therapy. White and light-grey bars represent theoretical and obtained values, respectively. Means and SD of three replicates are shown.

Abbreviations: PC: phosphatidylcholine.

**Supplementary Figure S8. Schematic representation of a Langmuir-Blodgett Balance experiment to study PS lateral structure and lipid composition under interfacial compression.**

PS was dispensed at the air-liquid interface, which was kept at the target temperatures along the experiment and checked by a probe overtime. The maximum variation in temperature was 1 °C. Material at the interface was transferred onto a glass slide, moving up the glass (5 mm/min) coupled with the interface compression (25 cm^2^/min) as illustrated by the red arrows. For epifluorescence experiments, around 30 μg of PS at 5 mg/mL was previously doped with BODIPY-PC (1% mol/mol, 1h at 37°C) and, once at the interface, transferred onto the glass slide before and after 10 cycles (65 cm^2^/min). The experiment was performed in darkness and domain structures were observed under an epifluorescence microscope. For lipidomic analysis, around 50 μg of PS at 5 mg/mL was dispensed at the interface, before transferring material. Lipids at the interface were rinsed with chloroform:methanol (2:1, v/v), dried by nitrogen (around 5’) and analyzed by LC-HRMS.

**Supplementary Table S1. Temperature-dependent changes in surfactant lipid species at the surface film, under compression of the interface.**

Lipidomic analysis of PS lipid species associated to the surface during compression of the interface (25 cm^2^/min) at 33 °C or 37 °C. 5 replicates were pool per condition. Each lipid species was normalized with respect of total PLs and reported as percentage.

Abbreviations: PC: phosphatidylcholine; PLs: phospholipids; PC-O: phosphatidylcholine plasmalogens; LPC: Lysophosphatidylcholine; PE: phosphatidylethanolamine; PG: phosphatidylglycerol; CHOL: cholesterol; DPPC: dipalmitoylphosphatidylcholine; POPC: palmitoyloleoylphosphatidylcholine; puPC: polyunsaturated phosphatidylcholine.

| **Lipids (% total PLs)** | **33 °C** | **37 °C** |
| --- | --- | --- |
| PC | 91.40 | 92.70 |
| PC-O | 1.84 | 1.27 |
| LPC | 1.79 | 0.97 |
| PE | 0.63 | 1.09 |
| PG | 4.34 | 3.96 |
| CHOL | 21.19 | 11.11 |
|  |  |  |
| **PC (% total PLs)** | **33 °C** | **37 °C** |
| PC 32:0, DPPC | 57.95 | 50.35 |
| PC 34:0 | 9.18 | 6.35 |
| PC 36:0 | 0.17 | 0.11 |
| PC 38:0 | 0.02 | 0.02 |
| PC 40:0 | 0.03 | 0.09 |
| PC (32:1) | 8.92 | 13.51 |
| PC (32:2) | 0.00 | 0.00 |
| PC (34:1), POPC | 10.70 | 13.00 |
| PC (34:2) | 2.28 | 4.96 |
| PC (36:1) | 1.15 | 1.72 |
| PC (36:2) | 0.88 | 2.06 |
| PC (36:3) | 0.06 | 0.41 |
| PC (36:4) | 0.01 | 0.05 |
| PC (38:1) | 0.01 | 0.02 |
| PC (38:2) | 0.02 | 0.04 |
| PC (38:3) | 0.00 | 0.00 |
| PC (38:4) | 0.00 | 0.00 |
| PC (40:1) | 0.02 | 0.02 |
| puPC (≥ 2 double bonds) | 3.24 | 7.52 |
|  |  |  |
| **PC-O (% total PLs)** | **33 °C** | **37 °C** |
| PC O-30:0 | 0.47 | 0.27 |
| PC O-32:0 | 1.18 | 0.76 |
| PC O-34:0 | 0.10 | 0.05 |
| PC O-32:1/P-32:0 | 0.02 | 0.06 |
| PC O-34:1/P-34:0 | **0.07** | **0.13** |
| PC O-34:2/P-34:1 | **0.00** | **0.01** |
|  |  |  |
| **LPC (% total PLs)** | **33 °C** | **37 °C** |
| LPC (16:0) | 1.33 | 0.59 |
| LPC (18:0) | 0.25 | 0.23 |
| LPC (16:1) | 0.03 | 0.00 |
| LPC (18:1) | 0.18 | 0.15 |
|  |  |  |
| **PE (% total PLs)** | **33 °C** | **37 °C** |
| PE (34:0) | 0.02 | 0.02 |
| PE (32:1) | 0.00 | 0.04 |
| PE (34:1) | 0.02 | 0.02 |
| PE (34:2) | 0.09 | 0.17 |
| PE (36:1) | 0.24 | 0.27 |
| PE (36:2) | 0.15 | 0.26 |
| PE (36:3) | 0.12 | 0.28 |
| PE (36:4) | 0.00 | 0.02 |
|  |  |  |
| **PG (% total PG)** | **33 °C** | **37 °C** |
| PG (32:0) | 1.42 | 1.09 |
| PG (34:0) | 0.05 | 0.06 |
| PG (32:1) | 0.35 | 0.43 |
| PG (34:1), POPG | 1.73 | 1.30 |
| PG (34:2) | 0.31 | 0.49 |
| PG (36:1) | 0.23 | 0.32 |
| PG (36:2) | 0.25 | 0.27 |
|  |  |  |
| **% total PLs** | **33 °C** | **37 °C** |
| saturated PLs | 72.18 | 59.99 |
| unsaturated PLs | 27.82 | 40.01 |

**Supplementary Table S2. Different concentrations and volumes used for the *in vitro* therapy experiments in CDS.** The different percentages of therapy used [(μg PLs therapy/μg PLs in inhibited PS)*100] are summarized together with the concentrations of materials and volumes dispensed on the top of the drop.

Abbreviations: DPPC: dipalmitoylphosphatidylcholine; POPG: palmitoyloleoylphosphatidylglicerol; SP-B: surfactant protein B; SP-C: surfactant protein C; PORα: poractant α; [ ]: concentration.

| **DPPC/POPG (65/35, w/w %) + SP-B/C (2% of total w)** | | | | | |
| --- | --- | --- | --- | --- | --- |
| 33 °C | | | 37 °C | | |
| Therapy (%) | Volume (nL) | [ ], mg/mL | Therapy (%) | Volume (nL) | [ ], mg/mL |
| 50 | 200 | 20 | 50 |  | |
| 80 | 150 | 40 | 80 | 250 | 40 |
| 100 |  | | 100 | 300 | 40 |
| **POR𝛂** | | | | | |
| 33°C | | | 37°C | | |
| Therapy (%) | Volume (nL) | [ ], mg/mL | Therapy (%) | Volume (nL) | [ ], mg/mL |
| 50 | 200 | 20 | 50 |  | |
| 80 | 150 | 40 | 80 |  | |
| 100 | 150 | 50 | 100 | 250 | 50 |
| 200 | 150 | 100 | 200 | 250 | 100 |

**Original and modified epifluorescence microscopy images used for the analysis**

**
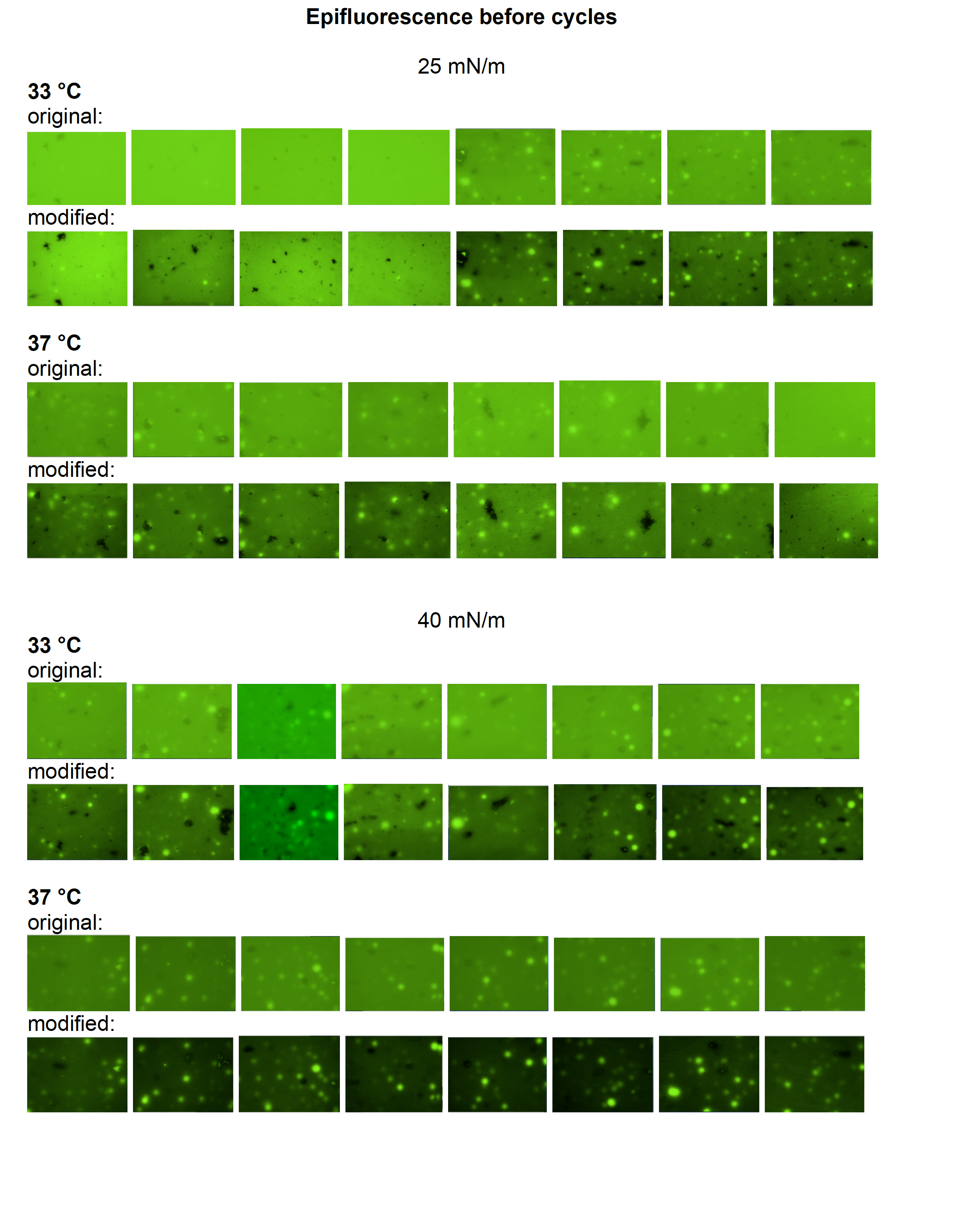
**

**
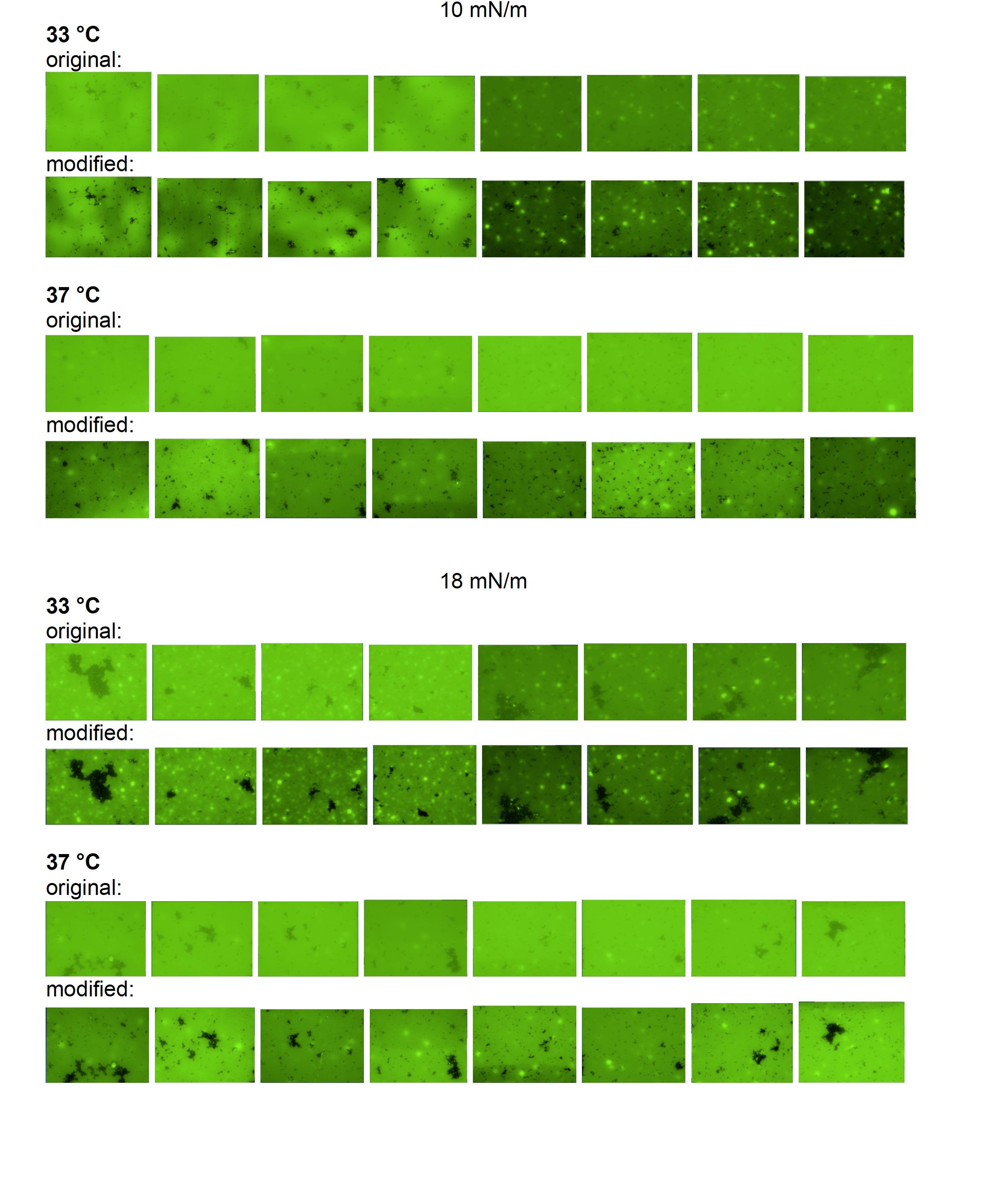
**


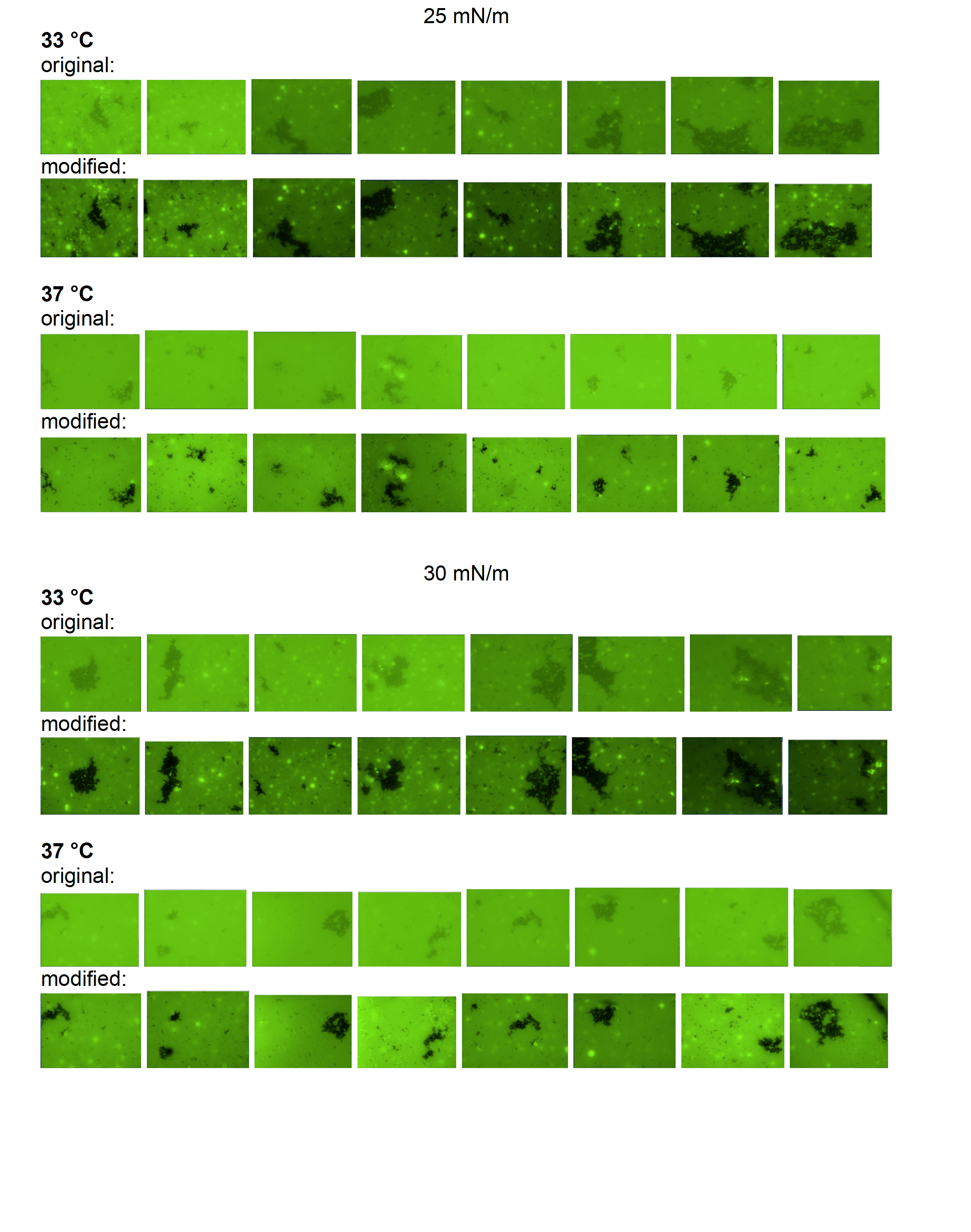

Supplement: Supplementary file 1 — Supplementary Information. [file 41598_2020_79025_MOESM1_ESM.docx]
